# Effect of sleep deprivation and stress on periodontal disease: A clinico-biochemical Study

**DOI:** 10.6026/973206300214459

**Published:** 2025-12-15

**Authors:** Pratisha Rahulkar, Laxman Boyapati, Abhigna Parikipandla, Aravind Kumar, Devarathnamma M.V, Pramod V, Miral Mehta

**Affiliations:** 1 Department of Periodontology, Meghna Institute of Dental Sciences, Nizamabad, Telangana, India; 2 Department of Dental surgery, Private Practitioner, Nizamabad, Telangana, India; 3 Department of Pediatric and Preventive Dentistry, Karnavati School of Dentistry, Karnavati University, Gandhinagar, Gujarat, India

**Keywords:** Sleep deprivation, stress, periodontal disease, biochemical assessment, clinical evaluation

## Abstract

Chronic stress and poor sleep are important lifestyle factors influencing that can influence the onset and progression of periodontal disease. Hence, a clinical biochemical study was conducted on 30 participants aged 25-50 years at Meghna Institute of Dental Sciences. Subjects were divided into three groups and FMBS, FMPS, PPD, CAL were recorded. Stress was measured using the 10-item Perceived Stress Scale (IPSS-10) and sleep quality was assessed using the Pittsburgh Sleep Quality Index (PSQI). It is found that periodontal disease is associated with stress, sleep deprivation and clinical indices such as CAL and PPD, while serum cortisol levels exhibited minimal variation across groups; the interplay of lifestyle factors highlights the need for a holistic approach to periodontal care.

## Background:

Chronic periodontitis (CP) is a multifactorial disease caused by microbial infection and host response, with dental biofilms providing the main cause of inflammation. These diseases are the sixth most frequent worldwide. Periodontitis' high economic cost and link with systemic diseases including diabetes, cardiovascular disease and Alzheimer's have made it a public health issue [14]. Periodontitis development and progression are influenced by various risk factors, both innate and acquired [20]. Notably, diabetes and smoking are key risk factors, while chronic and acute stress can impact periodontal health through biochemical or behavioural pathways. Stress triggers catabolic mechanisms by activation of the sympathetic autonomic nervous system and adrenocorticotropic axis and suppressing the parasympathetic system [1]. Immune cells manage, stimulate and prevent infection in periodontal tissues. Interleukins (IL-6, IL-1β, IL-8) and TNF-α play a role in phagocytic cell synthesis, regulating fibroblast and epithelial cell creation and repairing wounded sites [11]. During the normal healing process, the generation of these decreases considerably. Periodontal damage worsens in stressed periodontitis patients due to increased output [12]. Another modern issue is sleeping deprivation and every person needs to sleep, a complex biological process. Sleep deprivation affects cell repair, cognition, alterity, memory processing, hormone control, immunological function, depression risk, and cortisol and proinflammatory indicators. Insomnia can lead to increased levels of TNF-α, IL-6, peripheral leukocyte circulation and C-reactive protein (CRP) [9, 5]. Recently, investigators connected low sleep duration to systemic infections, citing host immunity experiments [13]. Since chronic periodontitis and sleep deprivation cause inflammation, few recent research have linked sleep length to periodontitis, with mixed results in India, the US and South Korea [15, 18, 19 and 22]. Stress and sleep deprivation are linked to chronic periodontitis, hence this study examined blood cortisol levels in the general population. Therefore, it is of interest to report the effect of sleep deprivation and stress on periodontal disease.

## Materials and Methods:

## Research design:

Clinical biochemical study was conducted between January 2024 to March 2024 in Meghna Institute of Dental Sciences Periodontics Department. The usual outpatient served as the source of 30 patients for the study. The goal of the study was explained to each participant and they all provided their consent. Male and female 25-50-year-olds with at least 20 teeth were studied. Participants who had periodontal therapy in the past year, pregnant women, smokers and those with systemic disorders were excluded. All subjects were examined by one examiner. Initial analysis of periodontal clinical parameters was done to determine which subjects were eligible for inclusion and exclusion. Then the following periodontal parameters were recorded: Full mouth plaque score (O'Leary TJ 1972) and bleeding score (Ainamo J, Bay I 1972), Probing pocket depth (PPD) and clinical attachment loss (CAL). Clinically study divided patients into three categories: GROUP 1 included subjects with healthy periodontium, i.e., no attachment loss or PPD of (<3 mm). GROUP 2 included subjects with chronic generalized gingivitis, i.e., without attachment loss and PPD of <3 mm. GROUP 3 included subjects with chronic periodontitis, i.e., the involvement of >30% sites with moderate to severe CAL >3 mm. All the subjects were provided with 10 item questionnaires to measure patients level of perceived stress (IPSS-10) and Pittsburgh sleep quality index (PSQI) questionnaire for assessing sleep quality.

## Perceived stress:

The 10-question instrument offered response options from 0 (never) to 4 (very often). Four questions (items 4, 5, 7 and 8) were positively stated (from 0 to 4). Sum scores were obtained by reversing positive item values and totalling all scores. The higher the score, the more stress was reported. The IPSS-10 score was divided into moderate/high (>13) and low felt stress (≤13) subgroups based on standards [22, 2].

## Pittsburgh sleep quality index:

The questionnaire scored seven sleep quality domains (0-3, 0-21): subjective sleep quality, sleep latency, sleep length, habitual sleep efficiency, sleep disruptions, use of sleeping drugs and daytime dysfunction. The scores of 5 and beyond were considered as "poor sleep quality," and those below 5 were considered as "good sleep quality" [3]. Subjects were instructed to visit the department between 8 AM and 10 AM to collect 2ml of blood to measure serum cortisol levels using the Cobas e 411 analysers with Electro Chemi Luminescence Assay. Sleep quality and stress were also assessed.

## Statistical analysis:

Using a Microsoft Excel Sheet, the data was saved in a computer. For each group of patient's data of blood serum and clinical parameters were analysed. Descriptive statistics were used and the mean and standard deviation obtained of each of the three groups. The Post Hoc test compared sleep quality to CAL and Cortisol. Pearsson's correlation compared the cortisol levels in all the three groups. The significance criterion was p< 0.05 for all.

## Results:

Table 1 (see PDF) shows socio-demographic, sleep quality, clinical indicators and correlations for three groups. The mean age increases from Group 1 (25.5 years) to Group 3 (45.9 years), with an average of 35.9 years. Group 2 (8 participants) are mostly male, while Groups 1 (9) and 3 (7) are female. Mostly students are present in group 1 (9), employees in Group 2 (6) and housewife and farmers occupation in Group 3. Table 2 (see PDF) represents the quality of sleep in three group of participants. Sleep quality is "fairly good" for the majority of participants (23) in all the three groups, with "very good" sleep being uncommon (3) and "fairly bad" more prevalent in Group 3. Table 3 (see PDF) represents the clinical parameters in all the three groups. There is a noticeable difference between the groups in terms of FMPS and PPD, with Group 1 has the lowest mean (1.280 ± 0.253) and 1.400 ± 0.516), followed by Group 2 (12.710 ± 6.984) and 1.900 ± 0.535) and Group 3 has the highest mean (38.350 ± 9.859) and (4.000 ± 0.747). Group 2 and Group 3 have considerably higher FMBS values than Group 1, which suggests that bleeding on probing is more noticeable in group 2 and 3. Group 1 has the highest cortisol levels, followed by Groups 2 and 3. This suggests that there may be a link between lower cortisol levels and higher clinical parameters like FMPS, PPD and FMBS in Groups 2 and 3. Clinical indicators indicate advanced periodontal problems in Group 3, with higher CAL (4.420 ± 0.728), FMPS (38.350 ± 9.859), PPD (4.000 ± 0.747) and FMBS (44.900 ± 15.234). Table 4 (see PDF) examines the correlation between cortisol levels, various sleep quality categories and Clinical Attachment Loss (CAL). With values ranging from -0.483 to -1.170, weak to moderate negative correlations are seen for CAL; however, none of these are statistically significant. Comparably, correlations among sleep quality categories for cortisol levels exhibit a wide range of variability, from 15.101 to -30.896, but none of these relationships are statistically significant. Overall, sleep quality, CAL and cortisol levels have some moderate to weak positive and negative associations, but these relationships are not significant enough to allow for the drawing of useful inferences from the data. Higher cortisol levels may be linked to a modest increase in CAL, as seen by the weak positive correlation found with CAL. Similar to CAL, PPD also has a weak positive correlation with cortisol levels, suggesting that elevated cortisol levels are associated with a slight increase in pocket depth. The trendline is almost flat, indicating a negligible relation between FMPS and cortisol levels. This implies that there is no significant association between cortisol levels and plaque scores. There is a negative correlation with FMBS, suggesting that somewhat lower bleeding scores may be linked to higher cortisol levels (Figure 1 - see PDF).

## Discussion:

Recent lifestyle changes have increased stress, causing sleep deprivation and periodontal infections. Stress and sleep deprivation may affect oral health biologically and behaviourally. This study assessed 30 people and recorded clinical parameters. Furuta *et al.* 2015 [16] carried out a longitudinal retrospective study investigating gender-related differences on periodontal health for an 8-year period. Females with pre-existing periodontal conditions were strong predictors of persistent periodontal disease. In contrast, in males, the relationship of inflammatory markers as well as with periodontal results was much weaker, which indicates females could react more intensively to involved immune mechanisms changing periodontal degradation. Correlating with the current study's findings, significant age and gender differences were observed, with p-values of 0.000 and 0.005, respectively, indicating that demographic factors notably influence periodontal health markers and potentially exacerbate age-related risks for periodontal deterioration. FMPS and FMBS were highest in Group 3 at 38.35 and 44.4, respectively, while CAL was 4.42, indicating a substantial difference between the three groups. Rohini *et al.* 2015 [17] stated that stress is correlated with plaque and periodontal disease which correlates with our study stating that there is an association of stress with periodontal disease. Sleep deprivation apparently has an unfavorable effect on health by suppressing immunity and altering metabolism. Alqaderi *et al.* 2020 [23] stated that individuals who sleep an average of more than seven hours a day and had never experienced sleep problems and those are not prone to having severe periodontal disease. Grover *et al.* 2015 [15] assessed sleep deprivation relative to chronic periodontal disease. PSQI was used to assess the sleep quality. Mean PSQI scores were significantly highest in the periodontitis group compared to the healthy and gingivitis groups. Conversely, in this present study, there was a trend toward near-significance in sleep scores between the groups (p = 0.049), where individuals with poor sleep quality showed mean CAL of 4.7, FMPS of 26.24 and FMBS of 31.06, which might be due to small sample size. With regard to the present study outcomes where sleep scores varied slightly between demographics, though less prominently than other factors, indicating stress and sleep as potential influencers of periodontal health. According to Marruganti *et al.* 2024 [25] stress and lack of sleep can multiply the prevalence and severity of periodontitis. Ge and Li, 2005 [6] stated that stress may be among the prominent risk indicators for periodontal disease in contrast to our study where there was a weak positive correlation between periodontitis and cortisol levels (p=0.188), signifying that stress markers are slightly varying and has less impact in periodontal outcomes. The course of periodontal disease can be determined through stress changing the reaction of the immune system of the body. There is also a potential that stress may lead to an elevated concentration of cortisol which may culminate in elevated levels of plaque accumulation, gingival inflammation as well as increased serum levels of pro-inflammatory cytokines like IL-6 and IL-10. There is a strong link between some biomarkers of stress and various clinical periodontal disease indicators. Therefore, psychological stress can cause direct effects on oral health. It therefore suggests that prolonged periods of stress could increase the chances of developing periodontal disease because it may interfere with immune functions and exacerbate inflammatory responses, making stress management part of oral health [8, 10].

Chronic stress has been shown to impair the body's ability to defend against periodontal pathogens. It may cause an immune response imbalance at both the cellular and humoral level, which would explain the rapid destruction of periodontal tissues. Stress also raises pro-inflammatory cytokines and bone-resorbing factors in gingival tissues, causing enhanced bone loss in periodontitis. Such stress-induced changes in inflammatory pathways may further accelerate the progression of periodontitis through glucocorticoid receptor signalling [4, 7]. Stress management and improved sleep quality can be an adjunctive measure in periodontal therapy. Chronic stress and inadequate sleep have negative effects on periodontal health due to their influence on compromising the immune system and causing inflammation. Mindfulness and other forms of relaxation can alleviate inflammation from stress and enhance healing in periodontal tissues. It is also important to mention that proper sleep is involved in host immunity preservation, which should be maintained at a good level for periodontal health. Stress management and sleep optimization used together with regular dental care significantly improve overall periodontal health [4]. Our study showed significance levels across a number of oral health measures, including the plaque score, bleeding score, mean clinical attachment level and pocket depth, which all demonstrated associations (p=0.000), paving the way toward suggestions that demographic factors and lifestyles might play an equally substantial role in explaining variance differences between groups. Dhinsa, 2021 [24] stated that CAL shows a statistically significant relationship with OHRQL and for other periodontal indicators like PPD and OHI-S while GI had only weak positive but non-significant association with OHRQL. Many limitations can be considered when drawing conclusions from the results of the study. The sample size in each group was relatively small, which limits generalization to other populations. Furthermore, the study's cross-sectional design rules out any causative links between stress, lack of sleep and periodontal diseases. This study also relies upon self-reported measures of evaluating sleep quality, as is the nature of this kind of subjective assessment and possibly biased or incorrect. Additionally, while biochemical markers such as cortisol have been included in this study, other potential confounders such as diet, smoking habits and medications were not controlled for in the assessment of oral health outcomes. Lastly, the study lacked research into the long-term consequences of stress and sleep deprivation on the periodontal conditions of adults, though useful knowledge would be generated in such chronic effects. The management of periodontal disease requires the assessment of lifestyle factors, including sleep and stress. Better sleep hygiene and stress reduction could promote the advancement of periodontitis. Smoking is a risk factor for periodontal disease and poor oral hygiene is and smoking is major contributors to its development [7]. Lifestyle factors such as body mass index (BMI), alcohol and sleeping behaviour have been associated with periodontitis, thus making it important to consider these factors in treatment planning. Healthy lifestyle measures like regular exercise are associated with more favourable periodontal conditions, indicating that comprehensive lifestyle management can benefit oral health. In addition, the management of these modifiable risk factors in conjunction with the conventional dental treatment can help reduce periodontal disease severity and improve the overall effectiveness of the treatment [26, 21].

## Conclusion:

Stress, sleep deprivation and factors like CAL and PPD are linked to periodontal disease. It also supports earlier researchers showing that stress can affect oral health by increasing cortisol and inflammation. The study's limited sample size, which includes smoking and obesity, should be identified while evaluating its value. Future longitudinal research with bigger, more representative samples may explore social support and stress coping methods to improve periodontal health management.
